# Development of Quantitative Real-time PCR Assays for Different Clades of “*Candidatus* Accumulibacter”

**DOI:** 10.1038/srep23993

**Published:** 2016-05-04

**Authors:** An Ni Zhang, Yanping Mao, Tong Zhang

**Affiliations:** 1 Environmental Biotechnology Laboratory, Department of Civil Engineering, The University of Hong Kong, Pokfulam Road, Hong Kong

## Abstract

We designed novel quantitative real-time polymerase chain reaction (qPCR) primers for the polyphosphate kinase 1 (*ppk1*) gene, targeting eight individual “*Candidatus* Accumulibacter” (referred to as Accumulibacter) clades. An evaluation of primer sets was conducted regarding the coverage, specificity, and PCR efficiency. (i) All primer sets were designed to cover all available sequences of the target clade. (ii) The phylogenetic analysis of the sequences retrieved from the qPCR products by each primer set demonstrated a high level of specificity. (iii) All calibration curves presented high PCR efficiencies in the range of 85–112% (R^2^ = 0.962–0.998). In addition, the possible interference of non-target amplicons was individually examined using the qPCR assay for 13 Accumulibacter clades, which were either undetected or showed negligible detection. With the primers designed by other research groups, a highly selective and sensitive qPCR-based method was developed to quantify all Accumulibacter clades, with the exception of Clade IE, in one assay, which enables more comprehensive insights into the community dynamics. The applicability to environmental samples was demonstrated by profiling the Accumulibacter clades in activated sludge samples of nine full-scale wastewater treatment plants.

Enhanced biological phosphorus removal (EBPR) has been highly utilized to remove phosphorous in wastewater treatment plants (WWTPs) around the world for nearly four decades. Polyphosphate accumulating organisms (PAOs) serve a major role in phosphorus removal by accumulating excess phosphate from wastewater inside their cell bodies. “*Candidatus* Accumulibacter” (referred to as Accumulibacter) has been identified as one of the primary unculturable PAOs in both full-scale WWTPs^1^,^2^,^3^ and lab-scale reactors^4^ that conduct EBPR.

The 16S ribosomal RNA (rRNA) method was used as the primary approach to identify the occurrence and phylogeny of Accumulibacter in EBPR systems, until their inability to partition Accumulibacter into ecotypes, owing to their high similarities, was proven. Then, polyphosphate kinase 1 (*ppk1*) gene encoding the major enzyme for catalysing polyphosphate synthesis was applied as a genetic marker to partition the Accumulibacter lineage into Type I and Type II (consists of four clades)^5^,^6^. Subsequently, quantitative real-time PCR (qPCR) primers that target Accumulibacter Type I and Clades IIA, IIB, IIC and IID, were designed and verified to differentiate divergence among Accumulibacter ecotypes^6^.

Knowledge of the phylogenetic and genetic diversity within the Accumulibacter lineage was then substantially expanded. In 2008, Peterson *et al.*^7^ subdivided the Accumulibacter Type I into five clades and discovered Clades IIE, IIF and IIG of Type II, based on the phylogenetic distance of the *ppk1* gene. The Accumulibacter lineage comprises five clades in Type I and nine clades in Type II, including the novel clades proposed by Mao *et al.*^8^.

In contrast with the rapid exploration inside the Accumulibacter lineage, the primer design work for recent discovered clades proceeded slowly. Only one primer set has been designed for Clade IIF^9^; no sets have been designed for the other identified clades (IA, IB, IC, ID, IE, IIE, IIG, IIH, and II-I). Retaining the coverage and sensitivity to the target clades has become a challenge for the previously designed qPCR assay. A recent study has revealed that the total Accumulibacter lineage in 10 of 18 WWTPs, as determined by former primer sets for the *ppk1* gene^6^, accounted for less than 50% of the total Accumulibacter determined by the Accumulibacter 16S rRNA gene^8^. The first reason for this result was that Clade IB was not covered by the primer set Acc-ppk1-763f and Acc-ppk1-1170r, which targets Accumulibacter Type I, and has therefore been described as unclassified^8^,^10^. Clade IB has been found to be the dominant Accumulibacter clade in an EBPR reactor, and a draft genome has been reconstructed^10^. The second reason was that the other dominant clades were not covered by the previous qPCR assay, which required new primer sets for identification and quantification. Thus, the primer design for these clades is required to study the entire profile of Accumulibacter clades in different environments.

The Accumulibacter *ppk1* gene sequences in public databases have been expanded and rapidly updated in recent years, providing sufficient reference to design primer sets with good coverage and specificity. Therefore, this study sought to develop a qPCR assay by primer design to comprehensively and specifically profile the distribution of Accumulibacter clades.

## Results

### Phylogenetic analysis of *ppk1* gene

**Figure 1:**
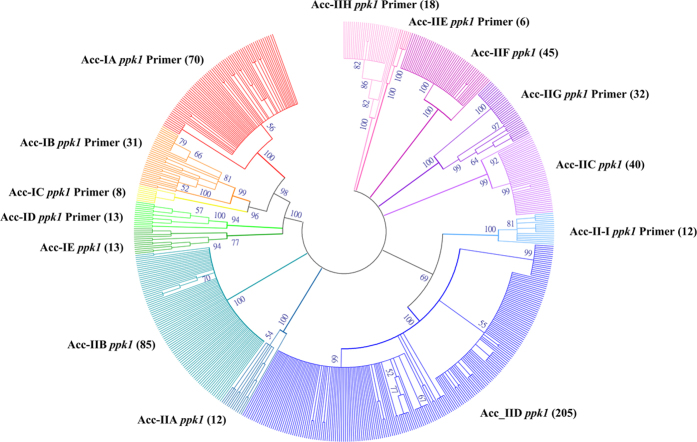
Condensed phylogenetic tree for 605 available Accumulibacter *ppk1* gene sequences, which were constructed by the maximum likelihood method using the Tamura-Nei model. Each clade was labelled by the previously assigned Acc (Accumulibacter) clade name^6^,^7^ and the primer name that was designed in this study. Bootstrap values are displayed as a percentage of 1000 replicates.

A database of 605 *ppk1* gene sequences of 14 known Accumulibacter clades (Supplementary Table S7) was retrieved from the NCBI database^7^,^8^,^9^,^11^. The maximum likelihood method with 1000 bootstrap replicates was used to construct a phylogenetic tree by MEGA (v. 6.06)^12^. The condensed tree with a bootstrap support value cutoff of 50%, is displayed to indicate the relationships among *ppk1* gene homologues (Fig. 1). The *ppk1* gene sequences were classified into five clades in Type I and nine clades in Type II, in accordance with the classification in previous studies^5^,^7^,^8^,^11^.

### Design of qPCR primer sets

Primer design was conducted using PRISE2^13^; it focused on nine clades without available primers, including Clades IA, IB, IC, ID, and IE in Type I and Clades IIE, IIG, IIH and II-I in Type II. The amplicon length, annealing temperature and inter/intra complimentary between forward primers and reverse primers were considered.

During primer design, sequences from the database were successively selected to represent different target clades, whereas all other sequences were selected to represent the non-target clades. The final primer sets were selected after considering the following criteria: (i) Both forward and reverse primers exactly match the majority of the target sequences and none of the non-target sequences, under very stringent settings (indicates high coverage and high specificity assuming no mismatch during the qPCR). (ii) Both forward and reverse primers match as few non-target sequences as possible, under very flexible settings (indicates high specificity considering potential mismatches during qPCR). (iii) Under flexible settings, the mismatches between the candidate primers and non-target sequences should be as far away from the 5′ end of the candidate primers as possible. The percentage of target sequences for both primers was calculated using the most stringent setting— 0MAM (mismatch allowance mechanism^13^) and the percentage of non-target sequences was calculated using all MAM settings (Supplementary Materials and Methods, Supplementary Table S4). As an example, the comparison between 3MAM and 1MAM, which showed 4.28% non-target sequences and 1.43% non-target sequences, respectively, aligned to Primer IB, indicated that the unspecific amplification of the 16 sequences of Clades ID and IE were more likely to be inhibited by distinctive 3′ end dissimilarity (Supplementary Table S1). However, Primer IB could not discriminate the other eight sequences of Clade IC at the terminal 3′ position, which were reflected by the comparison between 1MAM and 1MAM^*^. In comparison with other *in silico*-based primer design strategies, in our strategy, positional mismatches towards non-target sequences were considered to enhance the discriminatory power of the primer 3′ end. Thus, the specificity of the candidate primers was enhanced by introducing multiple MAM patterns to *in silico* evaluation, especially when many candidates satisfied the first two criteria. Criterion (iii) excluded candidate primers with mismatches at less discriminatory positions (central region or the primer 5′ end), which would certainly spare many efforts in *in vitro* examination. After *in silico* screening, two candidate primer sets were selected for each clade; the percentage of target sequences and the percentage of non-target sequences were 100% and 0%, respectively, when a perfect match was required during qPCR. According to these criteria, no primer set that targets the entire Type I or the entire Type II could be designed. In addition, the primer set Acc-ppk1-763f and Acc-ppk1-1170r, which targets Accumulibacter Type I^6^ partially covered the *ppk1* gene sequences that belong to Clade IA and one sequence of Clade IB (accession number EU432881) (Supplementary Table S6).

### Evaluation of the novel qPCR primer sets

Three mixtures of DNA samples from diverse sludge sources (18 WWTPs and an EBPR lab-scale reactor) were cocktailed as a DNA template to conduct PCR and qPCR using the candidate primer sets. Due to the rare appearance of Clade IE in the DNA template, primer sets that target Clade IE were not evaluated in this study. Amplicons of each primer set were cloned and sequenced for phylogenetic analysis. According to the assignment results, primer sets with amplicons that were affiliated only inside their target clades were selected for the efficiency evaluation. Two candidate primer sets for Clades IA, ID and IIE and one candidate primer set for Clades IC, IIF, IIG, IIH and II-I matched their theoretical specificities. Although the application of MAM patterns can increase the possibility of achieving specificity-qualified candidate primers, i.e., 11 of the 18 designed primers in this study, sequencing-based cross-amplification evaluation is necessary to examine the cross-amplification that is not completely predicted by *in silico* checking.

For Clade IB, the phylogenetic tree indicated that the qPCR products from two candidate primer sets were clustered within Clades IB, IC and ID. One primer set with relatively higher specificity was selected, and the qPCR condition was optimized by increasing annealing temperature. The qPCR products of two elevated annealing temperatures (57.0 °C and 58.0 °C) exhibited bright bands on agarose gel (Supplementary Fig. S9) and were cloned and sequenced. The consensus trees (Supplementary Fig. S2) indicated that the slight increase in annealing temperature significantly improved the specificity without a loss of coverage, on the basis of the diversity of two achieved clone libraries. To test the potential cross-amplification, the annealing temperature of qPCR using Primer-IB was set at 57.0 °C during future experiments.

The representative sequences obtained using the designed primer sets displayed high intra-clade dissimilarity, with identities to reference sequences from 93% to 100%. This result demonstrated the diversity and divergence within each clade in the DNA template. Although the true coverage of the Accumulibacter clades was not determined, the diversity of the sequences rendered confidence in the application of these primers to an extensive range of target sequences in the environment. This diversity also supported the specificity of the primers because numerous and diverse non-target sequences from all known clades, with the exception of Clade IE, were examined (Supplementary Table S2).

### PCR efficiency and interference of the designed primer sets

To evaluate the PCR efficiency, plasmids that contain amplicons of a specific clade were obtained using the candidate primers and previously designed primer sets^6^. The amplification effectiveness was evaluated by melting curves and six-point calibration curves in a ten-fold series. Six replicates for each plasmid copy number from 10^3^ to 10^8^ were applied within each assay. Only one primer set was selected according to its PCR efficiency. For all primer sets, a high PCR efficiency (85–112%) with a correlation coefficient in the range of 0.962–0.998 was achieved at the optimized annealing temperature and primer concentration (Table 1) to ensure the accuracy during quantification.

To explore the potential interference among different amplicons, we individually conducted qPCR with each primer set, using plasmids that contain amplicons of 13 Accumulibacter clades generated with primers listed in Supplementary Table S1, as DNA templates with the copy number of 10^7^. In this paper, a concept of “relative efficiency” was introduced to quantity the interference effect, which was calculated using the Ct value (referred to as the threshold cycle) with the following formula assuming a 100% amplification efficiency.


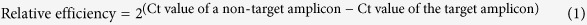


To define whether the existence of non-target amplicons might influence accurate quantification, we introduced an observed delay of less than ten cycles (referred to as the threshold cycle) as the criterion, which was equivalent to a relative efficiency of more than 0.1%^14^. As shown in Supplementary Table S3, 41.3% combinations had no interference (interference was not detected by qPCR, green nodes) and 57.7% had negligible interference (relative efficiency less than 2^−10^, yellow nodes), whereas only the combination of Primer-IC and Clade IIF exhibited slight interference (2^−9.95^, red node). The sum of nonspecific relative efficiencies for each primer set approximately indicated the total possibility of interference from non-target amplicons, which was significantly low—from 2^−17.0^ to 2^−8.5^.

### Application of the newly designed qPCR primer sets to quantify Accumulibacter clades in WWTPs

The occurrence of Accumulibacter clades in 18 full-scale WWTPs worldwide has previously been revealed^8^ by the qPCR-based method, with primer sets designed by He *et al.*^6^. More than 50% of the total Accumulibacter in ten of the 18 WWTPs may belong to some clades not targeted by previously designed primer sets. In this study, the newly designed primer sets were used to formulate an assay investigating the unclassified Accumulibacter in nine WWTPs, including the WWTPs of mainland China (CN-BJ-BX: Bei-Xiao-He, Beijing; CN-GZ-DT: Da-Tan-Sha, Guangzhou; CN-QD-TD: Tuan-Dao, Qingdao; CN-WH-LW: Long-Wang-Zui, Wuhan and CN-SH-MH: Min-Hang, Shanghai), Hong Kong (CN-HK-ST: Sha-Tin and CN-HK-SH: Shek-Wu-Hui), Singapore (SG-SG-UP: Ulu Pandan), and Japan (JP-A2O-TK: Tsukuba, WWTP using anaerobic/anoxic/aerobic (A/A/O) process). The abundance of one clade in the total bacterial population was calculated using the ratio of the cell number of Accumulibacter (assuming one *ppk*1 gene per cell) to the total bacteria cell number (based on 16S rRNA gene copy number). Both the *ppk1* and 16S rRNA gene copy numbers were obtained by qPCR^6^,^8^. The average *rrn* (rRNA) operon copy number per cell for each sample, except JP-A2O-TK, was calculated based on a reanalysis of their bacterial community structure by the QIIME pipeline^15^ (1.70v), using our previous data^16^ via Copyrighter-0.46^17^. For JP-A2O-TK, the *rrn* operon copy number per cell was represented by the average (2.04 copies per cell) of all samples.

**Figure 2:**
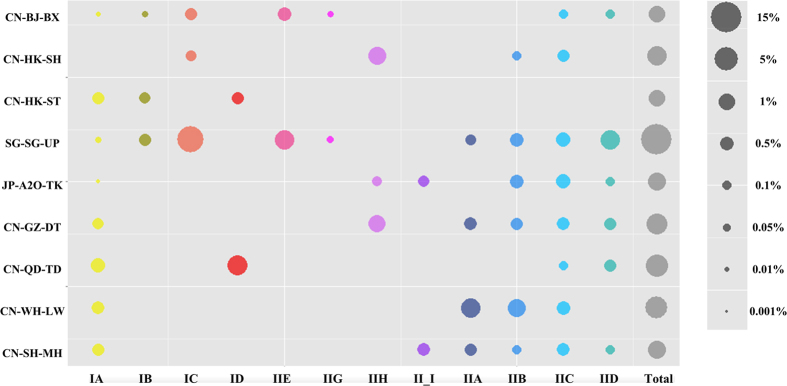
Estimated abundance of Accumulibacter clades relative to the total bacterial community in nine WWTPs. The size of the nodes represents the abundance of the indicated clade, as estimated by the Accumulibacter *ppk1* gene copy number to the bacterial 16S rRNA gene copy number^6^,^7^. The scale bar at the right side provides the reference value for the abundance.

The profile of different Accumulibacter clades in nine samples revealed that Accumulibacter existed in the SG-SG-UP with the highest abundance of 14.6% in the total bacterial community (Fig. 2). Among the 12 Accumulibacter clades, Clades IA, IIB and IIC appeared to be universally present and well adaptable, because they were detected in eight or nine geographically and operationally distinct WWTPs. The Accumulibacter lineage in SG-SG-UP and CN-BJ-BX exhibited a similar pattern and was dominated by Clades IC and IIE. Two novel clades—IIH and II-I—were discovered from CN-GZ-DT and JP-A2O-TK^8^, accounting for 1.3% and 0.3%, respectively, of the total bacterial community.

## Discussion

In this study, ten sets of qPCR primers were designed to target the Accumulibacter Clades IA, IB, IC, ID, IE, IIE, IIG, IIH, and II-I, which had no clade-specific primers available. The primer design method in this study introduced positional MAM^13^ settings to overcome the lack of valuing the position of mismatches in previous design strategies. The specificity and PCR efficiency of each candidate primer set were assessed and verified via PCR-cloning-sequencing, phylogenetic analysis, standard curves, and interference evaluation. Primer IE could not be evaluated due to limited sludge samples. Eight sets of primers (Table 1) were verified as highly selective and efficient. These primer sets exhibited 100% coverage of their target reference clades, 0% cross-amplification and high PCR efficiency (85–112%). The DNA template in this study consisted of spatially and temporally diverse sludge samples around the world, covering 13 known Accumulibacter clades and provided high confidence for the assessment results.

However, some limitations of this study remain. First, the primer set for Clade IE could not be assessed, owing to the rare occurrence of Clade IE in the samples. In addition, the coverage of the target clade and the diversity of the retrieved sequences were demonstrated only by *in silico* analysis as this diversity could not be validated via experiments. Only 20 clones were selected from the PCR products of each primer set for specificity evaluation. Finally, only three amplicons were selected from each clade for PCR efficiency evaluation; therefore, it may not have represented the diversity of samples in the environment.

With previously designed primer pairs^6^,^9^, we expanded the limited qPCR-based assay of the *ppk1* gene to a comprehensive assay to identify and quantify known Accumulibacter clades. The resolution of primers that target Type I was substantially improved by the individual quantification of Clades IA, IB, IC and ID. In addition, the profile of Accumulibacter Type II was comprehensively assessed, because all known clades were covered in the expanded assay. Therefore, this assay significantly resolves the problem in which a large portion of Accumulibacter clades could not be previously identified (Supplementary Table S5). However, the unclassified Accumulibacter abundance from 33.1% to 64.4% remains in 5 of 9 samples. The percentage of unidentified clades may have been from Clades IE and IIF, which were not quantified in this study, and Clade IIC, which was only partially covered by Primer IIC with 36.4% coverage (Supplementary Table S1) and the unknown Accumulibacter clades.

With more data provided by this new assay, potential directions for subsequent studies can be proposed: draft genome retrieving, gene expression dynamics and metabolic pathway analysis of dominant clades, especially for samples that were not previously reported^18^. For example, the results in this study demonstrated the high abundance of Clade IIE, i.e., 2.2% and 0.5% of the total bacterial community in SG-SG-UP samples and CN-BJ-BX samples, respectively, which indicates the significance and possibility of metagenomic analysis and genome binning of Clade IIE, which remains unknown.

## Materials and Methods

### Sludge samples collection

Three groups of sludge samples were collected as previously described^8^,^10^,^18^.

- (i) Temporal activated sludge (AS) samples were collected monthly from the middle of an aerobic (oxic) tank at the Shatin WWTP in Hong Kong from 2013 to 2014 (22°23′N 114°11′E). The tank, which was designed with a conventional anoxic/oxic (A/O) treatment process^18^, contained Accumulibacter Clades IB, IC and ID (Supplementary Table S2). The samples with a high abundance of Accumulibacter (>0.2% of total bacterial community) were selected based on our previous 16S rRNA gene analysis^18^.
- (ii) AS samples were collected from 18 globally distributed and full-scale WWTPs in Asia (Hong Kong, mainland China, Japan and Singapore), North America (Canada and the United States) and Europe (the United Kingdom)^8^,^16^, which contained Accumulibacter Clades IA, IB, IC, ID, IIE, IIG, IIH and II-I (Supplementary Table S2).
- (iii) PAO-enriched AS samples were collected from a well-performing EBPR batch reactor that was fed with acetate as the carbon source^10^ and contained Accumulibacter Clades IA, IB, IC, ID, IIE, IIG and IIH (Supplementary Table S2).

All samples were fixed by 50% ethanol at sampling and stored under −20 °C prior to DNA extraction.

### DNA extraction

Total DNA was extracted from 2.0 mL of the sludge samples using a FastDNA^®^ SPIN Kit for Soil (MP Biomedicals, Solon, OH). DNA purity at 260 nm and 280 nm was verified by a NanoDrop^®^ Spectrophotometer ND-100 (Thermo Fisher Scientific, USA), and the concentrations of the DNA samples were measured by a Qubit^®^ 2.0 Fluorometer (Invitrogen, Life Technology, USA).

The DNA samples were diluted to the same concentration and were completely mixed for each group. Three groups of mixed samples represented the AS of one full-scale WWTP in different operational conditions across a broad range of time, the AS of full-scale WWTPs from an extensive range of geographical locations, and the AS of the EBPR laboratory reactor. These samples provided high diversity of Accumulibacter lineage for 13 target clades.

### The *ppk1* gene database construction and phylogenetic analysis

A *ppk1* gene database was constructed with the reference sequences^7^,^8^,^9^,^11^, which were downloaded from the NCBI database. A total of 605 *ppk1* gene sequences from 14 known Accumulibacter clades were included. All sequences were aligned, and a phylogenetic tree was constructed using the maximum likelihood method by MEGA (v. 6.06)^12^. Bootstraps were calculated for 1000 replicates. The condensed tree was generated by establishing a cutoff bootstrap support value of 50% (Fig. 1).

### Primer design

Primer sets for qPCR were designed by PRISE2^13^ for Clades IA, IB, IC, ID and IE of Type I and for Clades IIE, IIG, IIH and II-I of Type II. Amplicons larger than 450 bp and primers with degenerate bases were avoided. The annealing temperature difference and inter/intra complimentary between forward primers and reverse primers were strictly controlled to ensure efficient amplification. Four different patterns of MAM were used to select highly specific primers, which are specified in the Supplementary Materials and Methods. Two candidate primer sets with 100% coverage of target clades and 0% coverage of non-target clades were selected for evaluation. A PCR-cloning-sequencing test was performed to ensure the specificity of each primer set, with the exception of Clade IE.

### qPCR conditions and *ppk1* gene clone library construction

Amplification of Accumulibacter *ppk1* gene fragments was conducted on a BioRad iCycler (v. 5.0, BioRad, Hercules, CA) in a 25 μL reaction volume. The qPCR was individually conducted on 5 ng mixed DNA samples as a template by primer sets for the *ppk1* gene (Table 1) and the primer set 341f/534r^6^ for 16S rRNA gene using SYBR Premix Dimer Eraser^TM^ (Takara, Japan). Thermal cycling and fluorescence detection were performed using the programme of initial denaturation at 95 °C for 30 s, followed by 40 cycles of denaturation at 94 °C for 5 s, annealing for 45 s, and extension at 72 °C for 30 s. The primer concentration and annealing temperature were adjusted to optimize PCR efficiency and specificity.

The amplicons of each group were purified by a quick-spin PCR Product Purification Kit (iNtRON, Korea) and equally mixed according to their concentrations. The mixed amplicons were verified by agarose gel and Bioanalyzer (2100, Agilent Technologies, Santa Clara, CA, USA). The amplicons with a single bright DNA band (or a single peak) at the expected size were selected for cloning using the pMD18-T vector (TaKaRa, Japan). Twenty clones were randomly selected from each library and sequenced to confirm the specificity of each primer set. The 20 sequences were searched against the *ppk1* gene database using BLASTN (v. 2.2.28+)^19^ and were aligned with the reference sequences to construct the phylogenetic tree (Supplementary Figs S1–S8). One primer set was selected for each clade considering the specificity and diversity within the target clade based on the phylogenetic results.

### Primer evaluation

Six-point calibration curves and melting curves were used to evaluate the efficiency by ten-fold series dilution. Regarding the reproducibility, the intra-assay variability was measured by conducting each single run using six replicates of amplicons with copy numbers from 10^3^ to 10^8^. The copy number of amplicons was calculated from the average molecular weight and the mass concentration^20^,^21^. A negative control without a DNA template was included to verify the possible contamination or primer-dimer formation in the PCR reactions.

The amplicons selected from each clade, with a copy number of 10^7^, were employed to evaluate the interference in the triplicate tests. For Clade IIC, two types of amplicons (IIC-1 and IIC-2) were obtained by the primer set that targets Acc-IIC *ppk1* and Acc-IIC *ppk1*, excluding OTU NS D3^6^.

### Application of the designed qPCR assay

DNA samples from nine globally distributed WWTPs were selected to profile different clades of Accumulibacter. These samples have been previously published and contain more than 50% unidentified Accumulibacter due to the limited availability of qPCR primers. In the qPCR reaction, 5 ng of genomic DNA was used as a template. To verify the intra-assay variability, the interference evaluation of the amplicons and the DNA samples were conducted in triplicate tests. The averages and standard deviations were calculated.

### Data analysis

The qPCR amplification efficiency was calculated based on the slope of the standard curve.


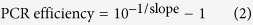


The qPCR amplification was considered valid only when the standard curves had a correlation coefficient greater than 0.96 and a PCR efficiency within the range 85–115%.

The abundance of Accumulibacter among the total bacterial community was normalized against the average 16S rRNA gene copy number per cell of each sample, which was calculated based on the bacterial composition by Copyrighter-0.46^17^. The bacterial composition was extracted from our previous study^16^. For JP-A2O-TK, we used the average of other AS samples.

### Nucleotide sequence accession numbers

The *ppk1* gene sequences obtained by Sanger sequencing have been deposited at GenBank under accession numbers of KT752523-KT752702. The accession numbers of amplicons applied in the PCR efficiency evaluation are as follows: KT752544-KT752546 (Clade IA), KT752630-KT752632 (Clade IB), KT752693-KT752695 (Clade IC), KT752606-KT752608 (Clade ID), KT752533-KT752535 (Clade IIE), KT752565-KT752567 (Clade IIG), KT752667-KT752669 (Clade IIH) and KT752590-KT752592 (Clade II-I). The accession numbers of amplicons applied in the interference assay are as follows: KT752544 (Clade IA), KT752630 (Clade IB), KT752693 (Clade IC), KT752606 (Clade ID), KP737891 (Clade IIA), KP738049 (IIB), KP737878 and KP738080 (IIC), KP738094 (IID), KT752533 (Clade IIE), KT752565 (Clade IIG), KT752667 (Clade IIH) and KT752590 (Clade II-I).

## Additional Information

**How to cite this article**: Zhang, A. N. *et al.* Development of Quantitative Real-time PCR Assays for Different Clades of “*Candidatus* Accumulibacter”. *Sci. Rep.* **6**, 23993; doi: 10.1038/srep23993 (2016).

## Supplementary Material

Supplementary Information

Supplementary Dataset 1

Supplementary Dataset 2

## Figures and Tables

**Table 1:** Information, qPCR conditions and performance of primers designed in this study.

		**Sequence (5**′**-3**′)	**Target**^b^	Ampliconsize (bp)	**Coverage**^c^	**Specificity (*****in silico***)^c^	**qPCR conditions**		qPCR performance(n **=** **6)**	
**Primer**^a^					Percentage oftarget sequences(0MAM^d^)	Percentage ofnon-target sequences(0MAM)	Primer concentration(nM)	*T*^a^(˚C)^e^	Standard curvecorrelationcoefficient	PCRefficiency^f^ **(%)**
Primer-IA	Acc-ppk1-974f	TGATGCGCGACAATCTCAAATTCAA	Clade IA	100%	0.0%	400	62.1	0.994 ± 0.002	108.1 ± 4.2	
	Acc-ppk1-1113r	AATGATCGGATTGAAGCTCTGGTAG								
Primer-IB*	Acc-ppk1-372f	TGAAGGCATTCGCTTCCT	Clade IB	282	100%	0.0%	400	58.0	0.982 ± 0.011	112.2 ± 8.7
	Acc-ppk1-653r	AAGCAGTATTCGCTGTC								
Primer-IC	Acc-ppk1-362f	AGCTGGCGAGTGAAGGCATTCG	Clade IC	397	100%	0.0%	350	65.5	0.986 ± 0.013	100.9 ± 4.3
	Acc-ppk1-758r	AACAGGTTGCTGTTGCGCGTGA								
Primer-ID	Acc-ppk1-634f	TGCGACAGCGAATACAG	Clade ID	215	100%	0.0%	400	58.1	0.984 ± 0.012	84.7 ± 7.9
	Acc-ppk1-848r	ACTTCGAGGCGGACG								
Primer-IIE	Acc-ppk1-757f	TTCGTGGACGAGGAAGA	Clade IIE	373	100%	0.0%	400	57.8	0.967 ± 0.016	100.0 ± 8.8
	Acc-ppk1-1129r	ATTGTTCGAGCAACTCGATG								
Primer-IIG	Acc-ppk1-410f	CCGAGCAACGCGAATGG	Clade IIG	105	100%	0.0%	400	62.1	0.979 ± 0.026	94.9 ± 8.8
	Acc-ppk1-514r	TGTTGAGTACGCGCGGGA								
Primer-IIH	Acc-ppk1-701f	ACTCCTTCGTATTCCTCTCT	Clade IIH	228	100%	0.0%	400	57.5	0.980 ± 0.007	93.2 ± 8.1
	Acc-ppk1-928r	TCATCGCTTCGGAGCA								
Primer-II-I	Acc-ppk1-688f	AGTGATTATGCTTTCGTCTTTC	Clade II-I	259	100%	0.0%	400	58.5	0.993 ± 0.004	102.2 ± 4.5
	Acc-ppk1-946r	TGAACTGTCCGAGCAGGA								

^a^Acc, Accumulibacter.

^b^Clade, Accumulibacter Clade.

^c^The percentage of target and non-target sequences was calculated against 605 available *ppk1* gene sequences of 14 known Accumulibacter clades.

^d^0MAM, no mismatch allowed, which indicated that a perfect match was required during the qPCR.

^e^*T*^*a*^, annealing temperature.

^f^PCR efficiency was estimated from the slope of the standard curve by equation (2). *During the evaluation, the annealing temperature of Primer-IB was set to 57 °C for potential cross-amplification detection. The standard curve of each primer set was examined by six-replicate tests.
