# Supplementary material for: Development of Quantitative Real-time PCR Assays for Different Clades of “Candidatus Accumulibacter”
Source: Sci Rep. 2016 May 4;6:23993. doi: 10.1038/srep23993 (PMC4855135; doi:10.1038/srep23993)
Supplement: Supplementary Information [file srep23993-s1.pdf]

Supporting Information for

Development of Quantitative Real-time PCR Assays for Different Clades of "*Candidatus* Accumulibacter"

Anni Zhang, Yanping Mao, Tong Zhang*

Environmental Biotechnology Laboratory, Department of Civil Engineering,

The University of Hong Kong, Pokfulam Road, Hong Kong

*Corresponding author: Tong Zhang, E-mail: zhangt@hku.hk; Tel.: +852 28578551;

Fax: +852 25595337.

Table and Figure Captions

Table S1. Characteristics of newly designed and previously reported qPCR primer sets.

Table S2. The occurrence of Accumulibacter clades in DNA samples detected by the qPCR assay using primer sets designed in this study.

Table S3. Interference evaluation of plasmids containing non-target amplicons.

Table S4. Positional MAM (mismatch allowance mechanism[1]) settings.

Table S5. Relative abundances (%) of indicated Accumulibacter clades within the Accumulibacter lineage by estimating the total abundance of the Accumulibacter lineage based on the qPCR assays targeting the 16S rRNA genes.

Table S6. Sequence alignment information (SAI) of Accumulibacter Type I sequences against Primer Acc-I *ppk1* (Acc-ppk1-763f and Acc-ppk1-1170r)[2]. The mismatches of alignment position between each reference sequence and forward/reverse primer sequences were highlighted in red.

Table S7 Accumulibacter *ppk1* gene database of 605 *ppk1* gene sequences of 14 known Accumulibacter clades retrieved from the NCBI database.

Figure S1. Phylogenetic tree indicating relatedness of "*Candidatus* Accumulibacter" *ppk1* gene sequences. Sequences from clone libraries

constructed with amplified fragments by Primer-IA were highlighted in bold. The *ppk1* gene sequences obtained in this study were compared with the reference ones downloaded from the NCBI database. The reference sequences of Accumulibacter Clade IA were underlined. Bootstrap values were calculated based on 500 replicates.

Figure S2. Phylogenetic tree indicating relatedness of "*Candidatus* Accumulibacter" *ppk1* gene sequences. Sequences from clone libraries constructed with amplified fragments by Primer-IB were highlighted in bold. Amplicon sequences displayed in the format of Times New Roman were achieved by qPCR under the annealing temperature (*Ta)* of 57.0 ˚C. Amplicon sequences displayed in the format of Arial Black were achieved under the annealing temperature (*Ta*) of 58.0 ˚C. The *ppk1* gene sequences obtained in this study were compared with the reference ones downloaded from the NCBI database. The reference sequences of Accumulibacter Clade IB were underlined. Bootstrap values were calculated based on 500 replicates.

Figure S3. Phylogenetic tree indicating relatedness of "*Candidatus* Accumulibacter" *ppk1* gene sequences. Sequences from clone libraries constructed with amplified fragments by Primer-IC were highlighted in

bold. The *ppk1* gene sequences obtained in this study were compared with the reference ones downloaded from the NCBI database. The reference sequences of Accumulibacter Clade IC were underlined. Bootstrap values were calculated based on 500 replicates.

Figure S4. Phylogenetic tree indicating relatedness of "*Candidatus* Accumulibacter" *ppk1* gene sequences. Sequences from clone libraries constructed with amplified fragments by Primer-ID were highlighted in bold. The *ppk1* gene sequences obtained in this study were compared with the reference ones downloaded from the NCBI database. The reference sequences of Accumulibacter Clade ID were underlined. Bootstrap values were calculated based on 500 replicates.

Figure S5. Phylogenetic tree indicating relatedness of "*Candidatus* Accumulibacter" *ppk1* gene sequences. Sequences from clone libraries constructed with amplified fragments by Primer-IIE were highlighted in bold. The *ppk1* gene sequences obtained in this study were compared with the reference ones downloaded from the NCBI database. The reference sequences of Accumulibacter Clade IIE were underlined. Bootstrap values were calculated based on 500 replicates.

Figure S6. Phylogenetic tree indicating relatedness of "*Candidatus* Accumulibacter" *ppk1* gene sequences. Sequences from clone libraries constructed with amplified fragments by Primer-IIG were highlighted in bold. The *ppk1* gene sequences obtained in this study were compared with the reference ones downloaded from the NCBI database. The reference sequences of Accumulibacter Clade IIG were underlined. Bootstrap values were calculated based on 500 replicates.

Figure S7. Phylogenetic tree indicating relatedness of "*Candidatus* Accumulibacter" *ppk1* gene sequences. Sequences from clone libraries constructed with amplified fragments by Primer-IIH were highlighted in bold. The *ppk1* gene sequences obtained in this study were compared with the reference ones downloaded from the NCBI database. The reference sequences of Accumulibacter Clade IIH were underlined. Bootstrap values were calculated based on 500 replicates.

Figure S8. Phylogenetic tree indicating relatedness of "*Candidatus* Accumulibacter" *ppk1* gene sequences. Sequences from clone libraries constructed with amplified fragments by Primer-II-I were highlighted in bold. The *ppk1* gene sequences obtained in this study were compared with the reference ones downloaded from the NCBI database. The reference

sequences of Accumulibacter Clade II-I were underlined. Bootstrap values were calculated based on 500 replicates.

Figure S9. Agarose gel electrophoresis images of amplified fragments using Primer-IB with different annealing temperature labeled under each well (56.0℃, 56.9℃, 58.3℃, 60.4℃, 63.5℃, 65.7℃, 67.2℃, 68.0℃). The qPCR products under the annealing temperature of 56.9℃ and 58.3℃ were purified for clone library construction.

Note：Table S6 and Table S7 are individual excel files (.xls).

Supplementary Materials and Methods

Positional MAM (mismatch allowance mechanism)[1] setting

A set of ideal primers should meet the following criteria. (i) Both forward and reverse primers exactly match the majority of the target sequences and none of the non-target sequences, under very stringent settings (indicates high coverage and high specificity assuming no mismatch during the qPCR). (ii) Both forward and reverse primers match as few non-target sequences as possible, under very flexible settings (indicates high specificity considering potential mismatches during qPCR). (iii) Under flexible settings, the mismatches between the candidate primers and non-target sequences should be as far away from the 5' end of the candidate primers as possible. The MAM sets limits on the accumulated number of mismatches per primer counting from the first base on the primer 3' end. Four patterns of MAM were used for simulating potential binding during qPCR between primers and sequences under different mismatch allowance (Table S4). The percentage of target or non-target sequences was defined as the portion of sequences that could bind to both forward and reverse primers under one MAM pattern. To specify, 3MAM allows at most one mismatch on the first three positions, two mismatches on the first twenty positions and three mismatches on all the positions; 1MAM specifies at most one mismatch on all positions, 1MAM* allows at most one mismatch on all positions with the exception of the first three positions and 0MAM allows no mismatch. To meet criterion (i), 0MAM was used to calculate the percentage of target sequences, where perfect match during qPCR is required. As to criterion (ii), the percentage of non-target sequences was calculated under all 4 MAM patterns (Supplementary Table S1) to simulate potential cross-amplification by allowing different mismatches during qPCR without reducing PCR amplification efficiency. Criterion (iii) was applied to value the mismatches towards non-target clades at different primer positions. This could be reflected by

comparing the difference between the results of 3MAM to 1MAM and 1MAM to 1MAM*. This difference represents the fraction of sequences with mismatches in the critical range[3] (last three bases from the 3' end of at least one primer), which are less likely to be amplified. The candidate primers were required to have as low non-target percentages under 3MAM and 1MAM as possible. If no primer can be designed with 0% non-target percentage under flexible settings, the difference between 3MAM to 1MAM, and 1MAM to 1MAM* should be as large as possible to increase discriminatory power at the primer 3' end, which may inhibit PCR more efficiently than the less sensitive central or 5' end positions[3].

Table S1. Characteristics of newly designed and previously reported qPCR primer sets.

Target[a]	Primer	Sequence (5'-3')	Coverage[b]	Specificity (*in silico*)[b]				Reference
			Percentage of target sequences	Percentage of non-target sequences				
			(0MAM)	(3MAM)	(1MAM)	(1MAM*)	(0MAM)	
Clade IA	Acc-ppk1-974f<br>Acc-ppk1-1113r	TGATGCGCGACAATCTCAAATTCAA<br>AATGATCGGATTGAAGCTCTGGTAG	100%	4.41%<br>IB(21), IE(2)	0.00%	0.00%	0.00%	This study
Clade IB	Acc-ppk1-372f<br>Acc-ppk1-653r	TGAAGGCATTCGCTTCCT<br>AAGCAGTATTCGCTGTC	100%	4.28%<br>IC(8), ID(11), IE(5)	1.43%<br>IC(8)	1.43%<br>IC(8)	0.00%	This study
Clade IC	Acc-ppk1-362f<br>Acc-ppk1-758r	AGCTGGCGAGTGAAGGCATTCG<br>AACAGGTTGCTGTTGCGCGTGA	100%	0.00%	0.00%	0.00%	0.00%	This study
Clade ID	Acc-ppk1-634f<br>Acc-ppk1-848r	TGCGACAGCGAATACAG<br>ACTTCGAGGCGGACG	100%	3.28%<br>IB(16), IC(1), IE(1)	0.00%	0.00%	0.00%	This study
Clade IIE	Acc-ppk1-757f<br>Acc-ppk1-1129r	TTCGTGGACGAGGAAGA<br>ATTGTTCGAGCAACTCGATG	100%	0.85%<br>ID(5)	0.00%	0.00%	0.00%	This study
Clade IIG	Acc-ppk1-410f<br>Acc-ppk1-514r	CCGAGCAACGCGAATGG<br>TGTTGAGTACGCGCGGGA	100%	6.25%<br>ID(7), IE(3), IID(18), IIE(6)	0.00%	0.00%	0.00%	This study
Clade IIH	Acc-ppk1-701f<br>Acc-ppk1-928r	ACTCCTTCGTATTCCTCTCT<br>TCATCGCTTCGGAGCA	100%	0.00%	0.00%	0.00%	0.00%	This study
Clade II-I	Acc-ppk1-688f<br>Acc-ppk1-946r	AGTGATTATGCTTTCGTCTTTC<br>TGAACTGTCCGAGCAGGA	100%	0.00%	0.00%	0.00%	0.00%	This study

Clade I	Acc-ppk1-763f	GACGAAGAAGCGGTCAAG	54.40%	46.90%	0.00%	0.00%	0.00%	2
	Acc-ppk1-1170r	AACGGTCATCTTGATGGC		IID(175), IIH(27)				
Clade IIA	Acc-ppk1-893f	AGTTCAATCTCACCGAGAGC	83.30%	23.60%	0.17%	0.17%	0.00%	2
	Acc-ppk1-997r	GGAACTTCAGGTCGTTGC		IID(118), IIG(11)	IID(1)	IID(1)		
Clade IIB	Acc-ppk1-870f	GATGACCCAGTTCCTGCTCG	95.30%	4.86%	2.37%	0.00%	0.00%	2
	Acc-ppk1-1002r	CGGCACGAACTTCAG		IIA(2), IIE(6), IIH(12), II-I(8)	IIH(12)			
Clade IIC	Acc-ppk1-254f	TCACCACCGACGGCAAGAC	36.40%	1.45%	1.45%	1.27%	0.00%	2
	Acc-ppk1-460r	CCGGCATGACTTCGCGGAAG		IIH(8)	IIH(8)	IIH(7)		
Clade IIC excluding OTU NS D3	Acc-ppk1-1123f	GAACAGTCCGCCAACGACC	36.40%	0.00%	0.00%	0.00%	0.00%	2
	Acc-ppk1-1376r	ACGATCATCAGCATCTTGGC						
Clade IID	Acc-ppk1-375f	GGGTATCCGTTTCCTCAAGCG	93.70%	3.88%	0.00%	0.00%	0.00%	2
	Acc-ppk1-522r	GAGGCTCTTGTTGAGTACACGC		IIA(9), IIE(6)				

[a]Clade represents Accumulibacter clade. [b]The percentage of target and non-target sequences were calculated against all 605 available *ppk1* gene sequences of 14 known Accumulibacter clades under four MAM patterns; The percentage of non-target sequences was calculated by simulating potential amplification considering mismatches during qPCR; The number of non-target sequences matching to each primer under one MAM pattern was listed below the percentage.

Table S2. The occurrence of accumulibacter clades in DNA samples detected by the qPCR assay using primer sets designed in this study.

DNA template	Target clade									
	IA	IB	IC	ID	IE	IIE	IIF	IIG	IIH	II-I
Group1	🟢	🔴	🔴	🔴	🟢	🟢	🟢	🟢	🟢	🟢
Group2	🔴	🔴	🔴	🔴	🟢	🔴	🔴	🔴	🔴	🔴
Group3	🔴	🔴	🔴	🔴	🟢	🔴	🔴	🔴	🔴	🟢

🔴 represents the target clade was detected by the qPCR assay and visualized by the gel image;

🟢 represents the target clade was not detected by the qPCR assay not reflected by the gel image.

Triplicate tests were conducted during qPCR experiments.

Group1: Temporal activated sludge (AS) samples collected monthly from Shatin WWTP, Hong Kong;

Group2: AS samples from 18 full-scale WWTPs across the world;

Group3: AS samples collected from a well-performing EBPR reactor.

Table S3. Interference evaluation of plasmids containing non-target amplicons[a].

Primer	Interference relative efficiency														Total non-specific relative efficiency
	IA	IB	IC	ID	IIA	IIB	IIC_1[c]	IIC_2[c]	IID	IIF	IIE	IIG	IIH	II_I	
Primer IA	🔵	🟡	🟡	🟡	🟡	🟡	🟡	🟡	🟡	🟡	🟡	🟡	🟡	🟡	$2^{-12.8}$
Primer IB[b]	🟡	🔵	🟡	🟢	🟡	🟡	🟡	🟡	🟡	🟡	🟡	🟡	🟡	🟡	$2^{-10.6}$
Primer IC	🟢	🟢	🔵	🟡	🟢	🟢	🟢	🟢	🟡	🔴	🟢	🟢	🟢	🟡	$2^{-8.5}$
Primer ID	🟢	🟡	🟢	🔵	🟢	🟢	🟢	🟡	🟢	🟡	🟡	🟢	🟡	🟡	$2^{-12.9}$
Primer IIE	🟢	🟢	🟢	🟡	🟢	🟢	🟢	🟢	🟢	🟡	🔵	🟢	🟡	🟢	$2^{-17.0}$
Primer IIG	🟡	🟢	🟡	🟡	🟡	🟡	🟢	🟢	🟡	🟡	🟡	🔵	🟢	🟡	$2^{-9.6}$
Primer IIH	🟡	🟡	🟢	🟡	🟡	🟡	🟡	🟡	🟡	🟡	🟡	🟡	🔵	🟢	$2^{-14.4}$
Primer II-I	🟢	🟡	🟢	🟡	🟢	🟢	🟢	🟢	🟢	🟢	🟡	🟢	🟢	🔵	$2^{-18.7}$

[a]Acc, Accumulibacter; represents relative efficiency = 1; represents relative efficiency = 2^{-10}-2^{-9}; represents relative efficiency < 2^{-10}; represents no effective amplification detected by qPCR assay. [b]During interference evaluation, the annealing temperature of Primer-IB was set at 57℃ for potential cross-amplification. [c]The plasmid of IIC_1 and IIC_2 were obtained from *ppk1* gene fragments amplified by the primer sets targeting Clade IIC and Clade IIC excluding OTU NS D3[2], respectively.

Table S4. Positional MAM (mismatch allowance mechanism)[1] settings[a].

Primer	5' end																								3' end
Position from 3' end	25	24	23	22	21	20	19	18	17	16	15	14	13	12	11	10	9	8	7	6	5	4	3	2	1
3MAM	**3**	**3**	**3**	**3**	**3**	**2**	**2**	**2**	**2**	**2**	**2**	**2**	**2**	**2**	**2**	**2**	**2**	**2**	**2**	**2**	**2**	**2**	**1**	**1**	**1**
1MAM	**1**	**1**	**1**	**1**	**1**	**1**	**1**	**1**	**1**	**1**	**1**	**1**	**1**	**1**	**1**	**1**	**1**	**1**	**1**	**1**	**1**	**1**	**1**	**1**	**1**
1MAM*	**1**	**1**	**1**	**1**	**1**	**1**	**1**	**1**	**1**	**1**	**1**	**1**	**1**	**1**	**1**	**1**	**1**	**1**	**1**	**1**	**1**	**1**	**0**	**0**	**0**
0MAM	**0**	**0**	**0**	**0**	**0**	**0**	**0**	**0**	**0**	**0**	**0**	**0**	**0**	**0**	**0**	**0**	**0**	**0**	**0**	**0**	**0**	**0**	**0**	**0**	**0**

[a]MAM limits on the accumulated number of mismatches counting from the first base on the 3’ end.

Table S5. Relative abundances (%) of indicated Accumulibacter clades within the Accumulibacter lineage by estimating the total abundance of the Accumulibacter lineage based on the qPCR assays targeting the 16S rRNA genes (n=3, relative standard deviation = 0.0%-28.1%).

Sample	IA	IB	IC	ID	IIE	IIG	IIH	II_I	IIA[a]	IIB[a]	IIC[a]	IID[a]	Unclassified clades	
													Previous study[4]	This Study
CN-BJ-BX	0.9	2.7	27.3	0.0	46.0	2.7	0.0	0.0	0.5	4.3	6.3	9.3	79.6	0.0
CN-HK-SH	0.0	0.0	6.5	0.0	0.0	0.0	45.2	0.0	0.0	4.4	10.6	0.3	84.7	33.1
CN-HK-ST	35.0	28.0	0.0	35.0	0.0	0.0	0.0	0.0	0.0	0.0	0.2	0.5	99.3	1.3
SG-SG-UP	0.2	2.1	58.5	0.0	15.2	0.3	0.0	0.0	1.4	3.4	4.3	14.6	76.3	0.0
JP-A2O-TK	0.3	0.0	0.0	0.0	0.0	0.0	8.7	16.7	0.0	30.9	39.6	3.7	9.1	0.1
CN-GZ-DT	5.3	0.0	0.0	0.0	0.0	0.0	27.7	0.0	7.5	6.3	8.7	6.0	71.5	38.5
CN-QD-TD	6.1	0.0	0.0	25.3	0.0	0.0	0.0	0.0	0.0	0.5	0.7	3.1	95.7	64.4
CN-WH-LW	5.7	0.0	0.0	0.0	0.0	0.0	0.0	0.0	25.6	17.2	6.1	0.0	51.1	45.4
CN-SH-MH	12.5	0.0	0.0	0.0	0.0	0.0	0.0	18.8	12.7	3.8	15.5	2.8	46.4	34.0

[a]The abundances of Clades IIA, IIB, IIC, IID and the original percentages of unclassified clades were achieved by previous study[4].

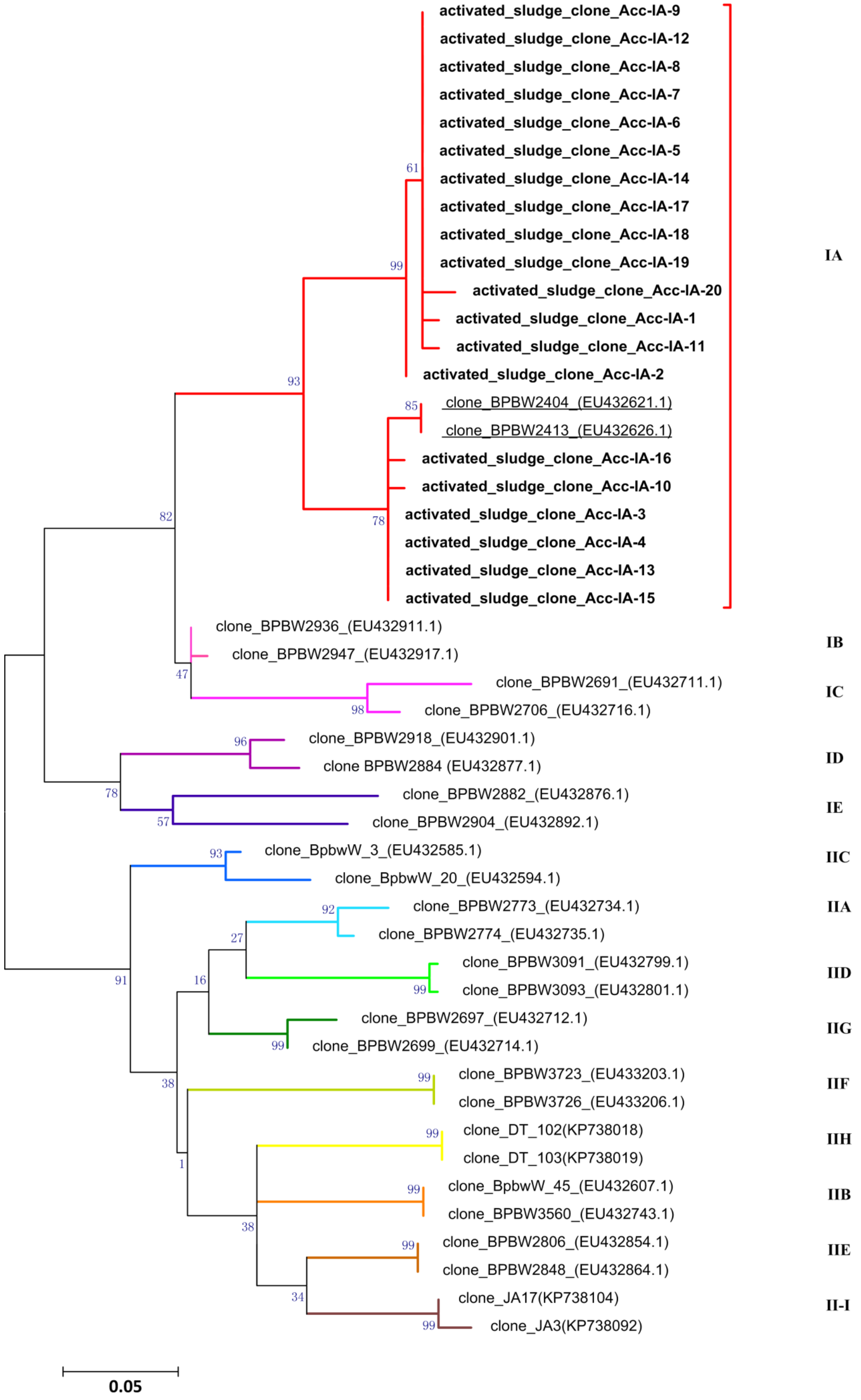
activated_sludge_clone_Acc-IA-9
activated_sludge_clone_Acc-IA-12
activated_sludge_clone_Acc-IA-8
activated_sludge_clone_Acc-IA-7
activated_sludge_clone_Acc-IA-6
activated_sludge_clone_Acc-IA-5
activated_sludge_clone_Acc-IA-14
activated_sludge_clone_Acc-IA-17
activated_sludge_clone_Acc-IA-18
activated_sludge_clone_Acc-IA-19
activated_sludge_clone_Acc-IA-20
activated_sludge_clone_Acc-IA-1
activated_sludge_clone_Acc-IA-11
activated_sludge_clone_Acc-IA-2
clone_BPBW2404_(EU432621.1)
clone_BPBW2413_(EU432626.1)
activated_sludge_clone_Acc-IA-16
activated_sludge_clone_Acc-IA-10
activated_sludge_clone_Acc-IA-3
activated_sludge_clone_Acc-IA-4
activated_sludge_clone_Acc-IA-13
activated_sludge_clone_Acc-IA-15
clone_BPBW2936_(EU432911.1)
clone_BPBW2947_(EU432917.1)
clone_BPBW2691_(EU432711.1)
clone_BPBW2706_(EU432716.1)
clone_BPBW2918_(EU432901.1)
clone BPBW2884 (EU432877.1)
clone_BPBW2882_(EU432876.1)
clone_BPBW2904_(EU432892.1)
clone_BpbwW_3_(EU432585.1)
clone_BpbwW_20_(EU432594.1)
clone_BPBW2773_(EU432734.1)
clone_BPBW2774_(EU432735.1)
clone_BPBW3091_(EU432799.1)
clone_BPBW3093_(EU432801.1)
clone_BPBW2697_(EU432712.1)
clone_BPBW2699_(EU432714.1)
clone_BPBW3723_(EU433203.1)
clone_BPBW3726_(EU433206.1)
clone_DT_102(KP738018)
clone_DT_103(KP738019)
clone_BpbwW_45_(EU432607.1)
clone_BPBW3560_(EU432743.1)
clone_BPBW2806_(EU432854.1)
clone_BPBW2848_(EU432864.1)
clone_JA17(KP738104)
clone_JA3(KP738092)
61
99
93
85
78
82
47
98
96
78
57
93
92
27
99
16
99
91
99
38
99
1
99
38
99
34
99
IA
IB
IC
ID
IE
IIC
IIA
IID
IIG
IIF
IIH
IIB
IIE
II-I
0.05

Figure S1. Phylogenetic tree indicating relatedness of "*Candidatus* Accumulibacter" *ppk1* gene sequences. Sequences from clone libraries constructed with amplified fragments by Primer-IA were highlighted in bold. The *ppk1* gene sequences obtained in this study were compared with the reference ones downloaded from the NCBI database. The reference sequences of Accumulibacter Clade IA were underlined. Bootstrap values were calculated based on 500 replicates.

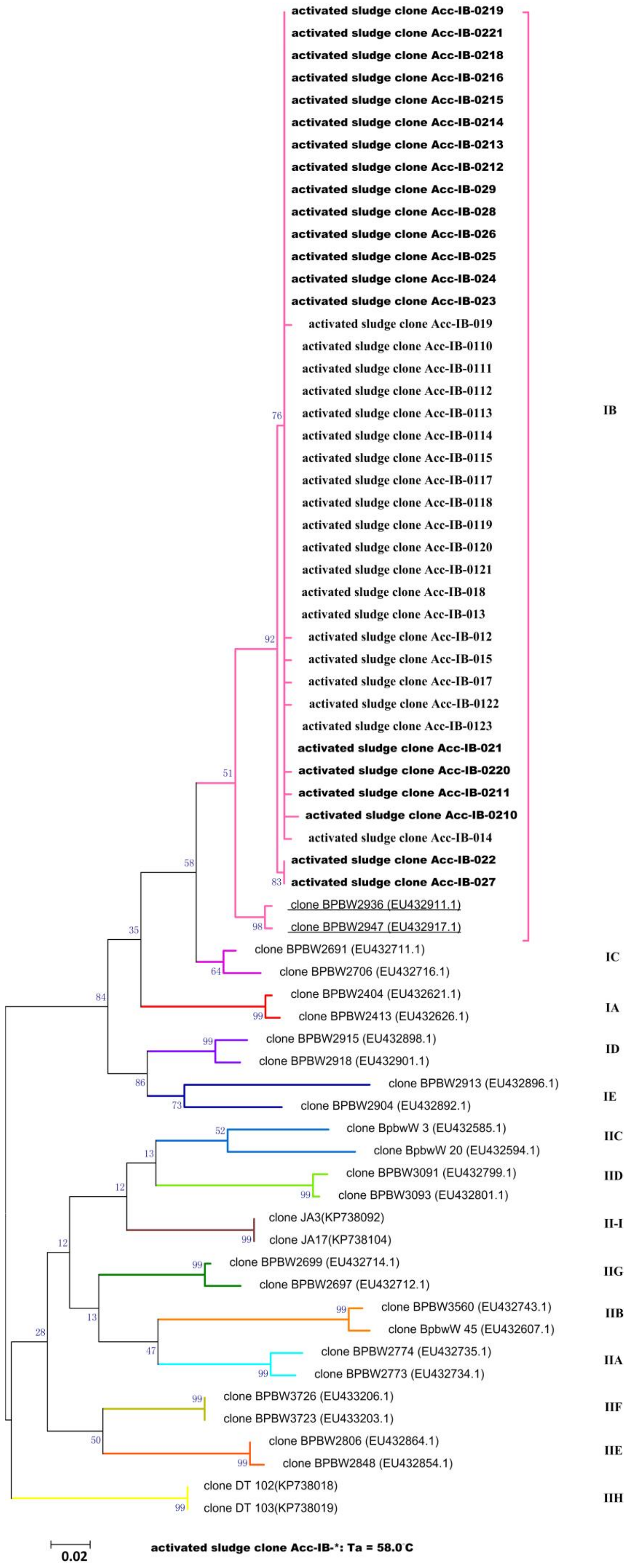

activated sludge clone Acc-IB-*: Ta = 58.0 C

activated sludge clone Acc-IB-*: Ta = 57.0°C

Figure S2. Phylogenetic tree indicating relatedness of "*Candidatus* Accumulibacter" *ppk1* gene sequences. Sequences from clone libraries constructed with amplified fragments by Primer-IB were highlighted in bold. Amplicon sequences displayed in the format of Times New Roman were achieved by qPCR under the annealing temperature (*Ta*) of 57.0 ˚C. Amplicon sequences displayed in the format of Arial Black were achieved under the annealing temperature (*Ta*) of 58.0 ˚C. The *ppk1* gene sequences obtained in this study were compared with the reference ones downloaded from the NCBI database. The reference sequences of Accumulibacter Clade IB were underlined. Bootstrap values were calculated based on 500 replicates.

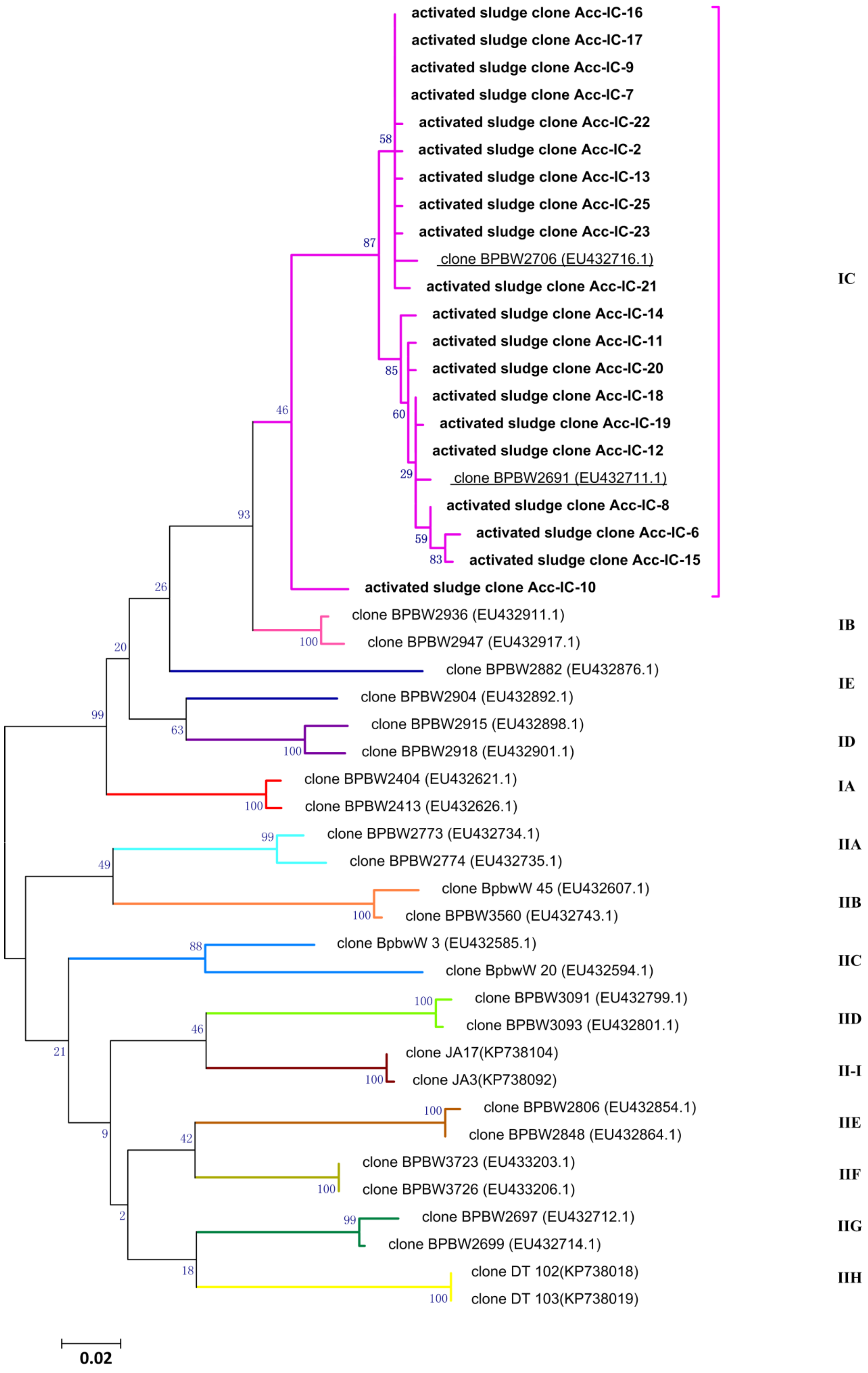
activated sludge clone Acc-IC-16
activated sludge clone Acc-IC-17
activated sludge clone Acc-IC-9
activated sludge clone Acc-IC-7
activated sludge clone Acc-IC-22
activated sludge clone Acc-IC-2
activated sludge clone Acc-IC-13
activated sludge clone Acc-IC-25
activated sludge clone Acc-IC-23
clone BPBW2706 (EU432716.1)
activated sludge clone Acc-IC-21
activated sludge clone Acc-IC-14
activated sludge clone Acc-IC-11
activated sludge clone Acc-IC-20
activated sludge clone Acc-IC-18
activated sludge clone Acc-IC-19
activated sludge clone Acc-IC-12
clone BPBW2691 (EU432711.1)
activated sludge clone Acc-IC-8
activated sludge clone Acc-IC-6
activated sludge clone Acc-IC-15
activated sludge clone Acc-IC-10
IC
clone BPBW2936 (EU432911.1)
clone BPBW2947 (EU432917.1)
IB
clone BPBW2882 (EU432876.1)
IE
clone BPBW2904 (EU432892.1)
clone BPBW2915 (EU432898.1)
clone BPBW2918 (EU432901.1)
ID
clone BPBW2404 (EU432621.1)
clone BPBW2413 (EU432626.1)
IA
clone BPBW2773 (EU432734.1)
clone BPBW2774 (EU432735.1)
IIA
clone BpbwW 45 (EU432607.1)
clone BPBW3560 (EU432743.1)
IIB
clone BpbwW 3 (EU432585.1)
clone BpbwW 20 (EU432594.1)
IIC
clone BPBW3091 (EU432799.1)
clone BPBW3093 (EU432801.1)
IID
clone JA17(KP738104)
clone JA3(KP738092)
II-I
clone BPBW2806 (EU432854.1)
clone BPBW2848 (EU432864.1)
IIE
clone BPBW3723 (EU433203.1)
clone BPBW3726 (EU433206.1)
IIF
clone BPBW2697 (EU432712.1)
clone BPBW2699 (EU432714.1)
IIG
clone DT 102(KP738018)
clone DT 103(KP738019)
IIH
0.02

Figure S3. Phylogenetic tree indicating relatedness of "*Candidatus* Accumulibacter" *ppk1* gene sequences. Sequences from clone libraries constructed with amplified fragments by Primer-IC were highlighted in bold. The *ppk1* gene sequences obtained in this study were compared with the reference ones downloaded from the NCBI database. The reference sequences of Accumulibacter Clade IC were underlined. Bootstrap values were calculated based on 500 replicates.

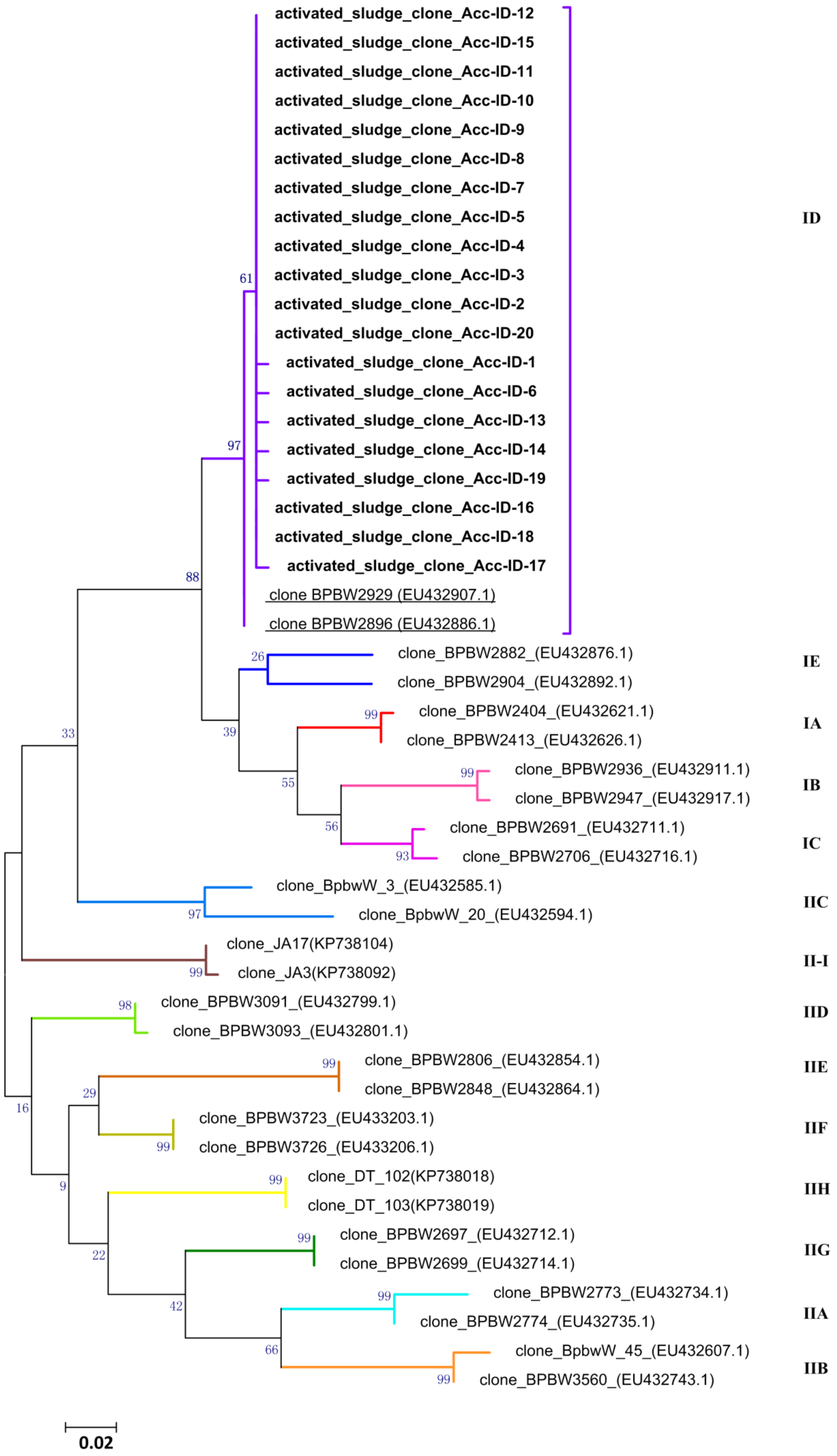
activated_sludge_clone_Acc-ID-12
activated_sludge_clone_Acc-ID-15
activated_sludge_clone_Acc-ID-11
activated_sludge_clone_Acc-ID-10
activated_sludge_clone_Acc-ID-9
activated_sludge_clone_Acc-ID-8
activated_sludge_clone_Acc-ID-7
activated_sludge_clone_Acc-ID-5
activated_sludge_clone_Acc-ID-4
activated_sludge_clone_Acc-ID-3
activated_sludge_clone_Acc-ID-2
activated_sludge_clone_Acc-ID-20
activated_sludge_clone_Acc-ID-1
activated_sludge_clone_Acc-ID-6
activated_sludge_clone_Acc-ID-13
activated_sludge_clone_Acc-ID-14
activated_sludge_clone_Acc-ID-19
activated_sludge_clone_Acc-ID-16
activated_sludge_clone_Acc-ID-18
activated_sludge_clone_Acc-ID-17
clone BPBW2929 (EU432907.1)
clone BPBW2896 (EU432886.1)
ID
clone_BPBW2882_(EU432876.1)
clone_BPBW2904_(EU432892.1)
IE
clone_BPBW2404_(EU432621.1)
clone_BPBW2413_(EU432626.1)
IA
clone_BPBW2936_(EU432911.1)
clone_BPBW2947_(EU432917.1)
IB
clone_BPBW2691_(EU432711.1)
clone_BPBW2706_(EU432716.1)
IC
clone_BpbwW_3_(EU432585.1)
clone_BpbwW_20_(EU432594.1)
IIC
clone_JA17(KP738104)
clone_JA3(KP738092)
II-I
clone_BPBW3091_(EU432799.1)
clone_BPBW3093_(EU432801.1)
IID
clone_BPBW2806_(EU432854.1)
clone_BPBW2848_(EU432864.1)
IIE
clone_BPBW3723_(EU433203.1)
clone_BPBW3726_(EU433206.1)
IIF
clone_DT_102(KP738018)
clone_DT_103(KP738019)
IIH
clone_BPBW2697_(EU432712.1)
clone_BPBW2699_(EU432714.1)
IIG
clone_BPBW2773_(EU432734.1)
clone_BPBW2774_(EU432735.1)
IIA
clone_BpbwW_45_(EU432607.1)
clone_BPBW3560_(EU432743.1)
IIB
61
97
88
26
99
39
33
99
55
56
93
97
99
98
99
29
16
99
99
9
99
22
42
99
66
99
0.02

Figure S4. Phylogenetic tree indicating relatedness of "*Candidatus* Accumulibacter" *ppk1* gene sequences. Sequences from clone libraries constructed with amplified fragments by Primer-ID were highlighted in bold. The *ppk1* gene sequences obtained in this study were compared with the reference ones downloaded from the NCBI database. The reference sequences of Accumulibacter Clade ID were underlined. Bootstrap values were calculated based on 500 replicates.

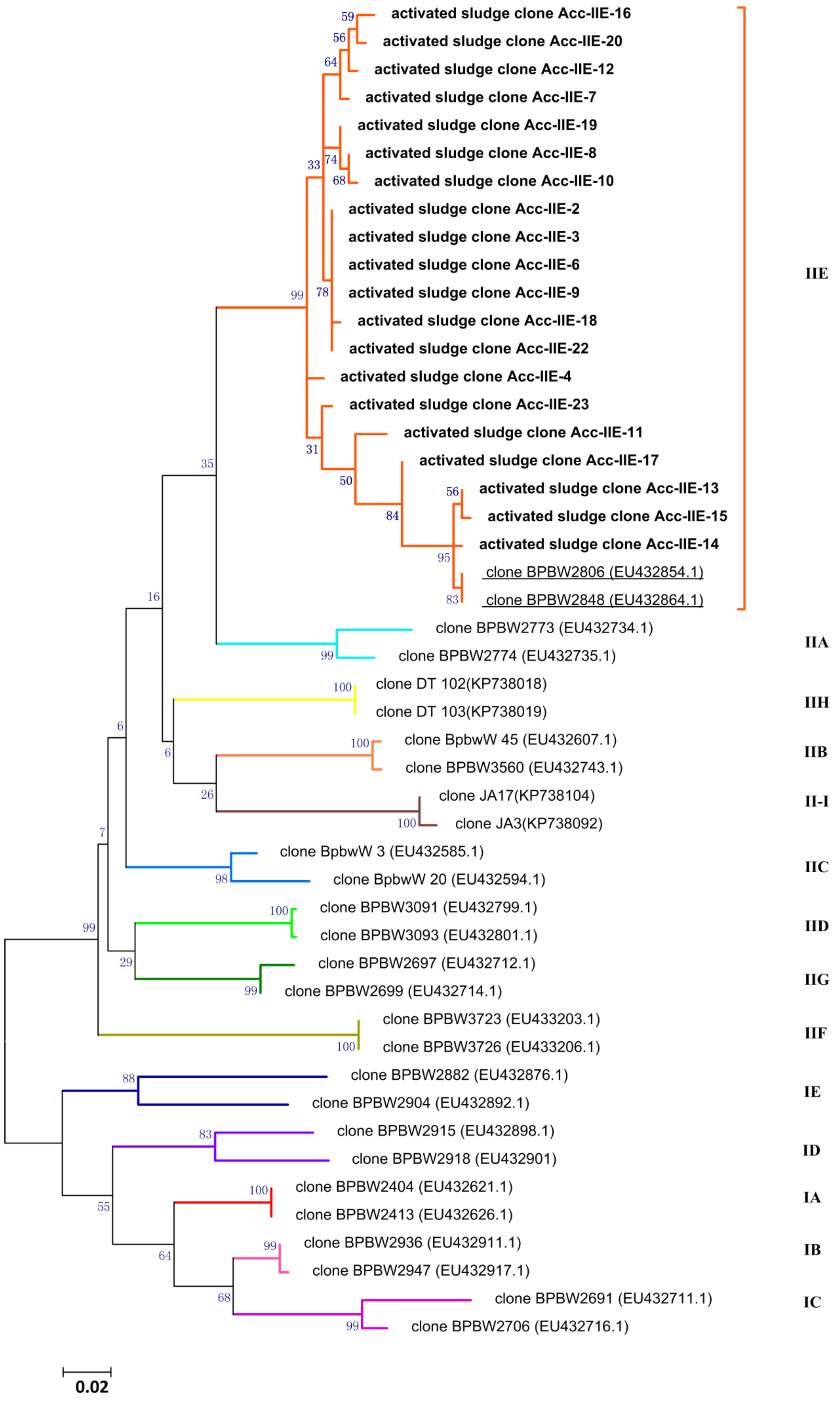
activated sludge clone Acc-IIE-16
activated sludge clone Acc-IIE-20
activated sludge clone Acc-IIE-12
activated sludge clone Acc-IIE-7
activated sludge clone Acc-IIE-19
activated sludge clone Acc-IIE-8
activated sludge clone Acc-IIE-10
activated sludge clone Acc-IIE-2
activated sludge clone Acc-IIE-3
activated sludge clone Acc-IIE-6
activated sludge clone Acc-IIE-9
activated sludge clone Acc-IIE-18
activated sludge clone Acc-IIE-22
activated sludge clone Acc-IIE-4
activated sludge clone Acc-IIE-23
activated sludge clone Acc-IIE-11
activated sludge clone Acc-IIE-17
activated sludge clone Acc-IIE-13
activated sludge clone Acc-IIE-15
activated sludge clone Acc-IIE-14
clone BPBW2806 (EU432854.1)
clone BPBW2848 (EU432864.1)
clone BPBW2773 (EU432734.1)
clone BPBW2774 (EU432735.1)
clone DT 102(KP738018)
clone DT 103(KP738019)
clone BpbwW 45 (EU432607.1)
clone BPBW3560 (EU432743.1)
clone JA17(KP738104)
clone JA3(KP738092)
clone BpbwW 3 (EU432585.1)
clone BpbwW 20 (EU432594.1)
clone BPBW3091 (EU432799.1)
clone BPBW3093 (EU432801.1)
clone BPBW2697 (EU432712.1)
clone BPBW2699 (EU432714.1)
clone BPBW3723 (EU433203.1)
clone BPBW3726 (EU433206.1)
clone BPBW2882 (EU432876.1)
clone BPBW2904 (EU432892.1)
clone BPBW2915 (EU432898.1)
clone BPBW2918 (EU432901)
clone BPBW2404 (EU432621.1)
clone BPBW2413 (EU432626.1)
clone BPBW2936 (EU432911.1)
clone BPBW2947 (EU432917.1)
clone BPBW2691 (EU432711.1)
clone BPBW2706 (EU432716.1)
IIE
IIA
IIH
IIB
II-I
IIC
IID
IIG
IIF
IE
ID
IA
IB
IC
0.02

Figure S5. Phylogenetic tree indicating relatedness of "*Candidatus* Accumulibacter" *ppk1* gene sequences. Sequences from clone libraries constructed with amplified fragments by Primer-IIE were highlighted in bold. The *ppk1* gene sequences obtained in this study were compared with the reference ones downloaded from the NCBI database. The reference sequences of Accumulibacter Clade IIE were underlined. Bootstrap values were calculated based on 500 replicates.

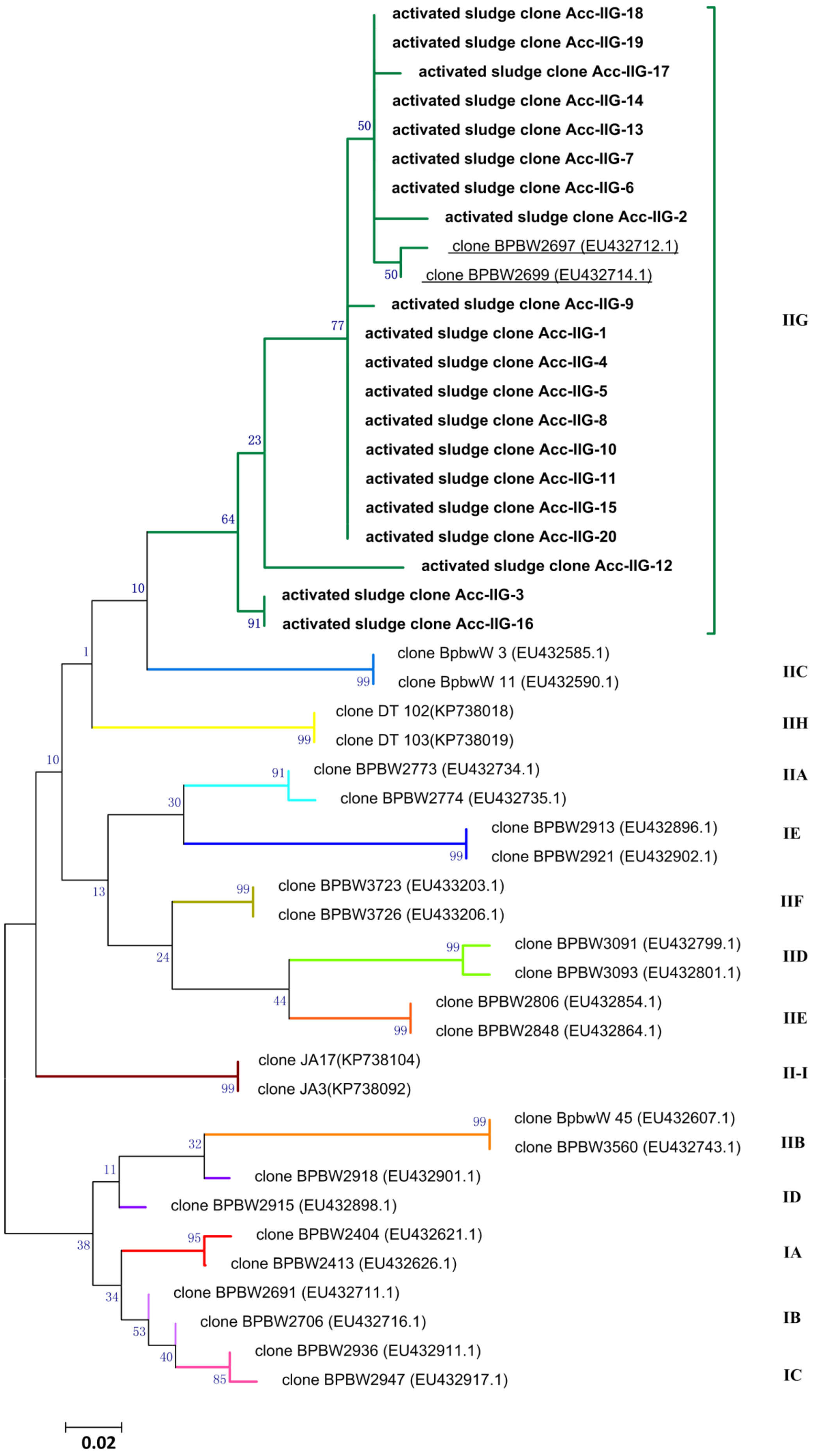
activated sludge clone Acc-IIG-18
activated sludge clone Acc-IIG-19
activated sludge clone Acc-IIG-17
activated sludge clone Acc-IIG-14
activated sludge clone Acc-IIG-13
activated sludge clone Acc-IIG-7
activated sludge clone Acc-IIG-6
activated sludge clone Acc-IIG-2
clone BPBW2697 (EU432712.1)
clone BPBW2699 (EU432714.1)
activated sludge clone Acc-IIG-9
activated sludge clone Acc-IIG-1
activated sludge clone Acc-IIG-4
activated sludge clone Acc-IIG-5
activated sludge clone Acc-IIG-8
activated sludge clone Acc-IIG-10
activated sludge clone Acc-IIG-11
activated sludge clone Acc-IIG-15
activated sludge clone Acc-IIG-20
activated sludge clone Acc-IIG-12
activated sludge clone Acc-IIG-3
activated sludge clone Acc-IIG-16
IIG
clone BpbwW 3 (EU432585.1)
clone BpbwW 11 (EU432590.1)
IIC
clone DT 102(KP738018)
clone DT 103(KP738019)
IIH
clone BPBW2773 (EU432734.1)
clone BPBW2774 (EU432735.1)
IIA
clone BPBW2913 (EU432896.1)
clone BPBW2921 (EU432902.1)
IE
clone BPBW3723 (EU433203.1)
clone BPBW3726 (EU433206.1)
IIF
clone BPBW3091 (EU432799.1)
clone BPBW3093 (EU432801.1)
IID
clone BPBW2806 (EU432854.1)
clone BPBW2848 (EU432864.1)
IIE
clone JA17(KP738104)
clone JA3(KP738092)
II-I
clone BpbwW 45 (EU432607.1)
clone BPBW3560 (EU432743.1)
IIB
clone BPBW2918 (EU432901.1)
clone BPBW2915 (EU432898.1)
ID
clone BPBW2404 (EU432621.1)
clone BPBW2413 (EU432626.1)
IA
clone BPBW2691 (EU432711.1)
clone BPBW2706 (EU432716.1)
IB
clone BPBW2936 (EU432911.1)
clone BPBW2947 (EU432917.1)
IC
0.02

Figure S6. Phylogenetic tree indicating relatedness of "*Candidatus* Accumulibacter" *ppk1* gene sequences. Sequences from clone libraries constructed with amplified fragments by Primer-IIG were highlighted in bold. The *ppk1* gene sequences obtained in this study were compared with the reference ones downloaded from the NCBI database. The reference sequences of Accumulibacter Clade IIG were underlined. Bootstrap values were calculated based on 500 replicates.

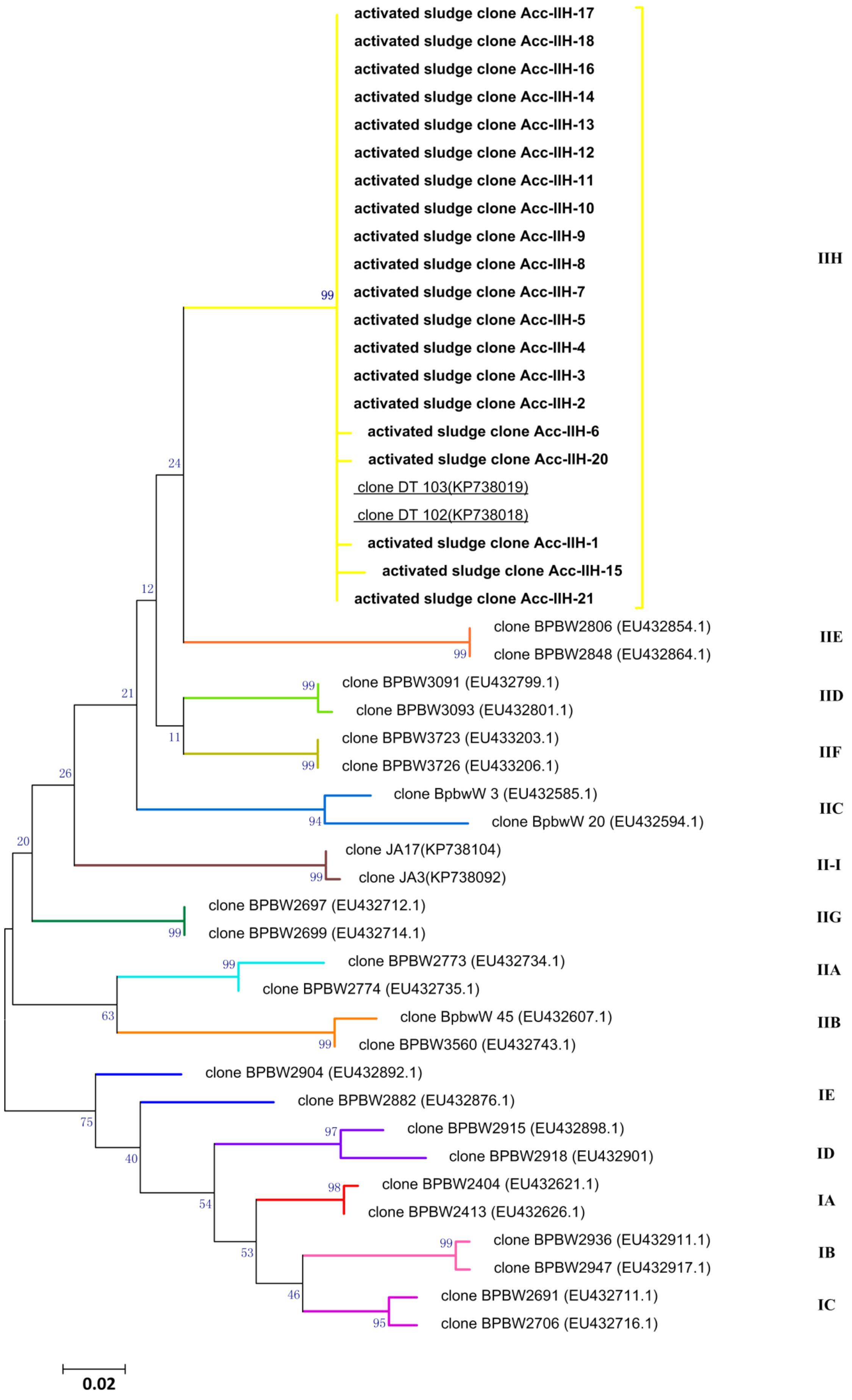
activated sludge clone Acc-IIH-17
activated sludge clone Acc-IIH-18
activated sludge clone Acc-IIH-16
activated sludge clone Acc-IIH-14
activated sludge clone Acc-IIH-13
activated sludge clone Acc-IIH-12
activated sludge clone Acc-IIH-11
activated sludge clone Acc-IIH-10
activated sludge clone Acc-IIH-9
activated sludge clone Acc-IIH-8
activated sludge clone Acc-IIH-7
activated sludge clone Acc-IIH-5
activated sludge clone Acc-IIH-4
activated sludge clone Acc-IIH-3
activated sludge clone Acc-IIH-2
activated sludge clone Acc-IIH-6
activated sludge clone Acc-IIH-20
clone DT 103(KP738019)
clone DT 102(KP738018)
activated sludge clone Acc-IIH-1
activated sludge clone Acc-IIH-15
activated sludge clone Acc-IIH-21
IIH
99
24
12
21
26
20
clone BPBW2806 (EU432854.1)
clone BPBW2848 (EU432864.1)
IIE
99
clone BPBW3091 (EU432799.1)
clone BPBW3093 (EU432801.1)
IID
99
11
clone BPBW3723 (EU433203.1)
clone BPBW3726 (EU433206.1)
IIF
99
clone BpbwW 3 (EU432585.1)
clone BpbwW 20 (EU432594.1)
IIC
94
clone JA17(KP738104)
clone JA3(KP738092)
II-I
99
clone BPBW2697 (EU432712.1)
clone BPBW2699 (EU432714.1)
IIG
99
clone BPBW2773 (EU432734.1)
clone BPBW2774 (EU432735.1)
IIA
99
63
clone BpbwW 45 (EU432607.1)
clone BPBW3560 (EU432743.1)
IIB
99
clone BPBW2904 (EU432892.1)
clone BPBW2882 (EU432876.1)
IE
75
40
clone BPBW2915 (EU432898.1)
clone BPBW2918 (EU432901)
ID
97
54
clone BPBW2404 (EU432621.1)
clone BPBW2413 (EU432626.1)
IA
98
53
clone BPBW2936 (EU432911.1)
clone BPBW2947 (EU432917.1)
IB
99
46
clone BPBW2691 (EU432711.1)
clone BPBW2706 (EU432716.1)
IC
95
0.02

Figure S7. Phylogenetic tree indicating relatedness of "*Candidatus* Accumulibacter" *ppk1* gene sequences. Sequences from clone libraries constructed with amplified fragments by Primer-IIH were highlighted in bold. The *ppk1* gene sequences obtained in this study were compared with the reference ones downloaded from the NCBI database. The reference sequences of Accumulibacter Clade IIH were underlined. Bootstrap values were calculated based on 500 replicates.

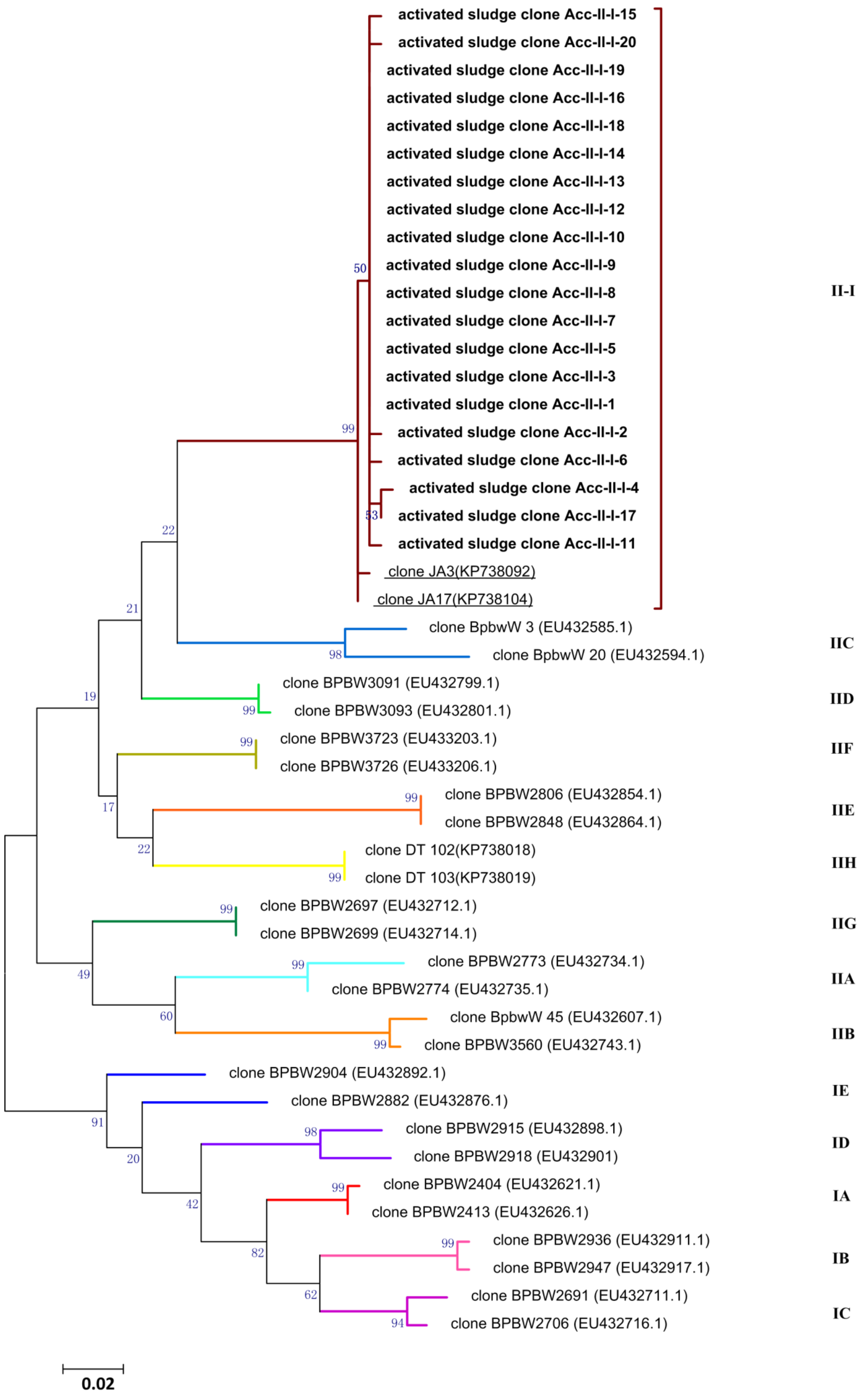
activated sludge clone Acc-II-I-15
activated sludge clone Acc-II-I-20
activated sludge clone Acc-II-I-19
activated sludge clone Acc-II-I-16
activated sludge clone Acc-II-I-18
activated sludge clone Acc-II-I-14
activated sludge clone Acc-II-I-13
activated sludge clone Acc-II-I-12
activated sludge clone Acc-II-I-10
activated sludge clone Acc-II-I-9
activated sludge clone Acc-II-I-8
activated sludge clone Acc-II-I-7
activated sludge clone Acc-II-I-5
activated sludge clone Acc-II-I-3
activated sludge clone Acc-II-I-1
activated sludge clone Acc-II-I-2
activated sludge clone Acc-II-I-6
activated sludge clone Acc-II-I-4
activated sludge clone Acc-II-I-17
activated sludge clone Acc-II-I-11
clone JA3(KP738092)
clone JA17(KP738104)
II-I
clone BpbwW 3 (EU432585.1)
clone BpbwW 20 (EU432594.1)
IIC
clone BPBW3091 (EU432799.1)
clone BPBW3093 (EU432801.1)
IID
clone BPBW3723 (EU433203.1)
clone BPBW3726 (EU433206.1)
IIF
clone BPBW2806 (EU432854.1)
clone BPBW2848 (EU432864.1)
IIE
clone DT 102(KP738018)
clone DT 103(KP738019)
IIH
clone BPBW2697 (EU432712.1)
clone BPBW2699 (EU432714.1)
IIG
clone BPBW2773 (EU432734.1)
clone BPBW2774 (EU432735.1)
IIA
clone BpbwW 45 (EU432607.1)
clone BPBW3560 (EU432743.1)
IIB
clone BPBW2904 (EU432892.1)
IE
clone BPBW2882 (EU432876.1)
clone BPBW2915 (EU432898.1)
clone BPBW2918 (EU432901)
ID
clone BPBW2404 (EU432621.1)
clone BPBW2413 (EU432626.1)
IA
clone BPBW2936 (EU432911.1)
clone BPBW2947 (EU432917.1)
IB
clone BPBW2691 (EU432711.1)
clone BPBW2706 (EU432716.1)
IC
0.02

Figure S8. Phylogenetic tree indicating relatedness of "*Candidatus* Accumulibacter" *ppk1* gene sequences. Sequences from clone libraries constructed with amplified fragments by Primer-II-I were highlighted in bold. The *ppk1* gene sequences obtained in this study were compared with the reference ones downloaded from the NCBI database. The reference sequences of Accumulibacter Clade II-I were underlined. Bootstrap values were calculated based on 500 replicates.

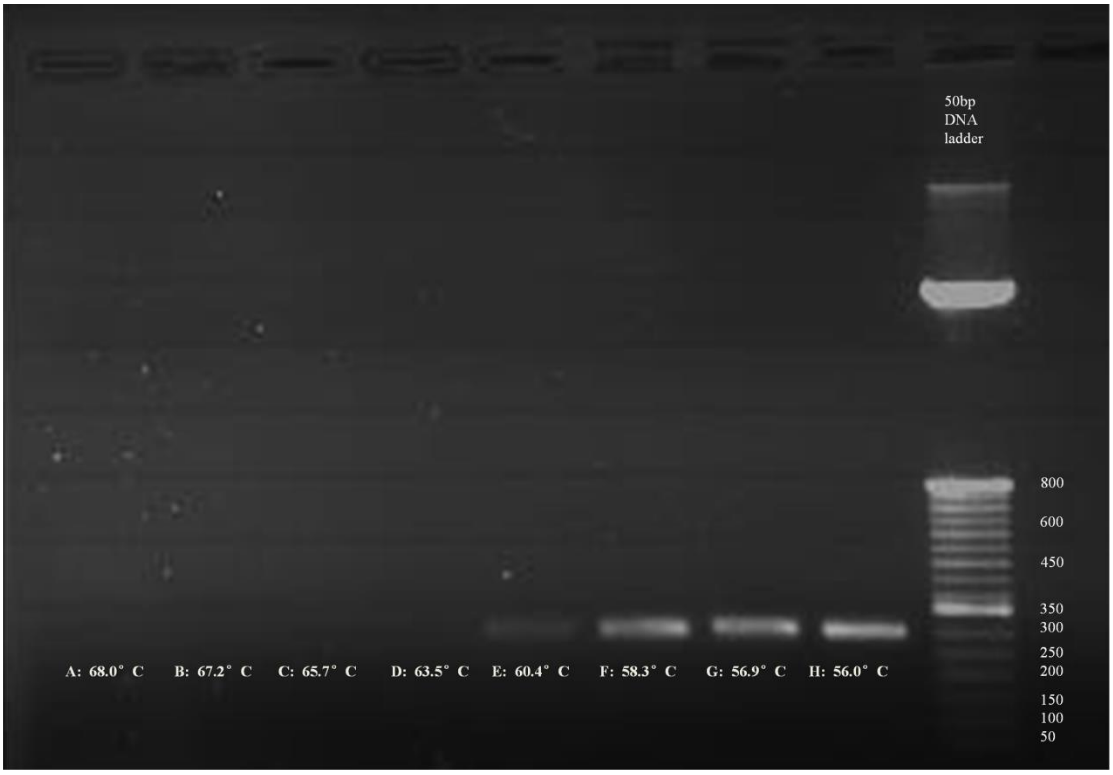

Figure S9. Agarose gel electrophoresis images of amplified fragments using Primer-IB with different annealing temperature labeled under each well (56.0℃, 56.9℃, 58.3℃, 60.4℃, 63.5℃, 65.7℃, 67.2℃, 68.0℃). The qPCR products under the annealing temperature of 56.9℃ and 58.3℃ were purified for clone library construction.

References

1. Huang, Y. T., Yang, J.-i., Chrobak, M. and Borneman, J. PRISE2: Software for designing sequence-selective PCR primers and probes. *BMC Bioinformatics*. **15,** 317 (2014).

2. He, S., Gall, D. L. and McMahon, K. D. "*Candidatus* Accumulibacter" population structure in enhanced biological phosphorus removal sludges as revealed by polyphosphate kinase genes. *Appl. Environ. Microbiol*. **73,** 5865-74 (2007).

3. Petruska, J. et al. Comparison between DNA melting thermodynamics and DNA polymerase fidelity. *Proc. Natl. Acad. Sci.* **85**, 6252-6256 (1988).

4. Mao, Y., Graham, D. W., Tamaki, H. and Zhang, T. Dominant and novel clades of *Candidatus* Accumulibacter phosphatis in 18 globally distributed full-scale wastewater treatment plants. *Sci. Rep*. **5,** 11857 (2015).
